# Clarifying the concept of avoidable emergency department attendance

**DOI:** 10.1177/1355819620921894

**Published:** 2020-06-10

**Authors:** Beth Parkinson, Rachel Meacock, Katherine Checkland, Matt Sutton

**Affiliations:** 1 PhD student in Health Economics, Health Organisation, Policy and Economics, The University of Manchester, UK; 2 Senior Lecturer in Health Economics, Health Organisation, Policy and Economics, The University of Manchester, UK; 3 Professor of Health Policy and Primary Care, Health Organisation, Policy and Economics, The University of Manchester, UK; 4 Professor of Health Economics, Health Organisation, Policy and Economics, School of Health Sciences, The University of Manchester, UK

**Keywords:** avoidable, emergency care, emergency department, health policy, preventable

## Abstract

Emergency department attendances are rising in several countries. Many of the policies aimed at reducing emergency department attendances are based on the assumption that a proportion of current utilization is ‘avoidable’ and therefore could be reduced. In considering how to achieve this aim, it is important to first understand the problem. In this essay, we review the literature on the concept and identification of avoidable emergency department attendances in England. We identified three areas of inconsistency surrounding avoidable emergency department attendances: the terminology, the underlying definition, and the method used to identify avoidable attendances. We offer a more nuanced definition which may better support action to reduce emergency department activity. Recognizing that there are different types of undesirable utilization which vary by underlying causes and potential solutions will aid policy makers in identifying areas where policies targeting reductions in emergency department attendances would best be directed.

## Introduction

Rising demand for emergency care is a common feature of health systems internationally. Increases in attendances at emergency departments (EDs) have placed considerable pressure on services. Evidence suggests that the resulting crowding in EDs has adverse effects including delayed treatment, increases in the number of patients leaving without being seen and adverse health outcomes including increased mortality.^1^ Reducing ED attendances is therefore a priority for health systems around the globe.

In England, there were 23.4 million ED attendances in 2016/2017, a 22% increase from 2007/2008.^2^ The associated annual cost was approximately £3 billion, representing 5.8% of the total cost of all acute services and 2.8% of total health expenditure in England.^3^ Both the National Health Service (NHS) Long-Term Plan and the Five-Year Forward View highlight reducing pressure on emergency hospital services as a policy priority.^4^,^5^ The Five-Year Forward View outlined a number of interventions aimed at easing pressure in EDs including front-door clinical streaming, improving the NHS 111 urgent medical concerns helpline, and improving access to General Practitioner appointments.^7^ In addition to these commitments, the NHS Long-Term Plan details further improvements to pre-hospital urgent care including a multidisciplinary Clinical Assessment Service to help patients navigate services, and Urgent Treatment Centres.^5^

Many of these policies are based on the assumption that a proportion of current utilization is ‘avoidable’ and could be reduced, thereby easing pressure on the system and saving money without compromising population health outcomes. In considering how to achieve this aim, it is important to first understand the problem. There is no standardized definition of an ‘avoidable’ ED attendance.^6^ Many proposed policy solutions are underpinned by assumptions rather than evidence about why patients attend the ED. Furthermore, avoidable attendances are often treated as a homogenous category and there is limited consideration of the different drivers of attendances. If progress is to be made in this area, a more nuanced approach is required.

In this article, we aim to review and critique the concept of avoidable ED attendances. We then offer a refined definition intended to be more useful for both policy and research, and discuss the next steps needed to operationalize this refined definition.

## Overview of the literature on defining avoidable ED attendances

The concept of avoidable utilization will differ by health system, as it is determined by the institutional arrangements, payment systems and availability of other services. We therefore focus on definitions describing the concept of avoidable attendances that have been used in previous studies of ED attendances in England and discuss how the concept may differ for other health systems. We identified and considered three systematic reviews concerning avoidable attendances published since 2005.^6–8^ We then ran forward citation searches from these reviews and their included papers to identify and analyse studies that were more recent.

We identified three areas of inconsistency surrounding the concept of avoidable ED attendances. First, there are differences in the terminology used. Second, there are differences in the definitions used. Third, there are differences in the way that the concept has been operationalized in terms of the method used to identify avoidable attendances in empirical studies.

In addition to the term ‘avoidable’, the terms ‘preventable’, ‘non-urgent’, ‘unnecessary’, ‘inappropriate’ and ‘primary care problems’ have also been used to describe the concept of ED attendances that should not have occurred from the perspective of the health system. Whilst these terms might appear to refer to different things, in practice the underlying concepts overlap, and in most studies relate to the potential for the patient to be managed elsewhere, most often in primary care. Other definitions include patients with trivial, low priority or low urgency problems,^9^ patients unlikely to require admission^10^ or patients who do not the need specialized services of an ED.^8^

In the absence of a standardized definition, a number of different methods and criteria have been used to operationalize the concept of avoidable ED attendances. These include the professional opinion of clinicians, by examination of the patient whilst in the department^11^ or retrospective clinical notes review,^12^ retrospectively applying pre-determined criteria to attendance data,^10^ or classifying patients into a pre-determined triage category.^13^ The clinical criteria used to determine avoidable attendances are often developed in consultation with a panel of experts, including ED clinicians, GPs and patient representatives; however, in some studies the origin of the definitions is not clear.

### The reported prevalence of avoidable ED attendances

Policies targeting reductions in ED attendances often cite figures on the number of attendances that are ‘avoidable’. For example, the 2013 Urgent and Emergency Care Review for England stated that ‘40 per cent of patients who attend an A&E department are discharged requiring no treatment. Many of these individuals could have been helped just as well closer to home’^14^ (p. 19). More recently, the NHS England Five-Year Forward View states that ‘between 1.5 and 3 million people who come to A&E each year could have their needs addressed in other parts of the urgent care system’^5^ (p. 14). NHS Digital, the national information and technology partner to the NHS, produce reports showing the number of ‘non-urgent’ ED attendances in England, suggesting that 16.1% of ED attendances occurring between April 2015 and December 2017 were ‘non-urgent’.^15^

International systematic reviews of the literature have also highlighted large variability in estimates, with between 10% and 90% of ED attendances classified as ‘inappropriate’,^6^ and 4.8% to 90% classified as ‘non-urgent’.^7^ The inconsistency in prevalence estimates across both policy statements and the academic literature is largely due to the lack of a standardized definition of avoidable attendances.^6^ Moreover, definitions are often very broad, encompassing types of attendance which are driven by very different causes. This limits opportunities for tailoring appropriate policy responses. For example, the definition used by NHS Digital to underpin their estimates is: ‘First attendance with some recorded treatments or investigations all of which may have been reasonably provided by a GP, followed by discharge home or to GP care’^15^ (p. 1). This definition encompasses both minor illnesses (which could potentially be dealt with by primary care providers) and self-limiting problems (which did not require any medical care at all), whilst not capturing serious problems arising because of earlier deficiencies in the medical care provided.

## Clarifying the concepts of avoidable ED attendances

A more nuanced understanding of the various causes leading to an ED attendance is needed if policy interventions are to be effective in achieving reductions in avoidable attendances. We suggest that there are at least three distinct categories of avoidable ED attendances, classified based on the care that was required. Classifying attendances according to the care that was required highlights the aspects of the system which could be targeted by policy interventions, and so focuses on potential solutions.

These concepts are defined from the perspective of the health care system, as this is the perspective taken by policy interventions. We recognize that categories derived from other perspectives (such as that of the patient) may be different. The categories are designed to be of use to health system planners to quantify the current magnitude of different types of avoidable attendances, identify areas where policies targeting reductions in ED attendances would best be directed, and to monitor the progress of any such policies.

### Clinically divertible attendances

The first subset describes patients who, given their present health care needs, would have been more appropriately treated elsewhere in the healthcare system. These are attendances during which a patient did require clinical attention of some form, but not the specialist services of the ED. This would suggest that a patient has accessed care which is more specialized in terms of the complexity and technicality of investigations and treatments available than was required to meet their healthcare needs. In many cases, this may impose higher costs on the system.

The most appropriate service that these patients should have been diverted to will depend upon whether their presenting healthcare needs were urgent. Non-urgent patients could have been diverted to routine primary care appointments, whilst those presenting with urgent care needs may have received more appropriate care from an urgent or out-of-hours primary care service.

Divertible patients did require clinical attention. These types of attendances are therefore likely to be responsive to increasing the availability of other health services, such as general practices, or ensuring that low-intensity urgent services are available and clearly signposted. It might also be necessary to increase patient confidence in these alternative services.^16^

### Clinically preventable attendances

The second subset of avoidable attendances describes situations when the presenting health care needs of the patient did require the type of specialized urgent care only available in the ED, but whose attendance could have potentially been prevented with earlier intervention or better management of their condition. Whilst this phenomenon has received attention when patients require hospital admission due to potentially preventable episodes,^17^ the preventability of ED attendances has received less attention.

The concept of ambulatory care-sensitive conditions (ACSCs) was developed to describe medical conditions where good out-of-hospital care, usually primary care, could prevent the need for hospital admission.^17^ These include acute conditions such as dehydration and gastroenteritis, where timely and effective care in the preceding few days could have stopped the condition deteriorating; chronic conditions such as asthma or diabetes, where good quality care over a longer period could have prevented flare-ups; and vaccine-preventable conditions.^17^

There is a need to extend this concept to consider potentially preventable ED attendances. ACSCs have been used to identify potentially preventable ED attendances in a number of US-based studies.^18^,^19^ However, a recent study in Germany suggests that further research is needed towards the development of a suitable and specific ACSC definition for research in the ED setting.^20^ Preventable attendances may be responsive to improving access to and the quality of primary care services. However, the appropriate response will vary depending on whether targeting acute, chronic or vaccine preventable conditions.

### Clinically unnecessary attendances

The third subset of avoidable attendances describes situations where a patient did not require any clinical care. Presenting at any urgent clinical care services would therefore be deemed ‘clinically unnecessary’ from the perspective of the healthcare system. This category is likely to be the most heterogeneous. This would range from patients whose condition only requires self-care to individuals presenting with complex social needs, who may require other, non-clinical, forms of care (e.g. social services).

In contrast to clinically divertible attendances, clinically unnecessary attendances are unlikely to be responsive to changes to the provision of health care services. Reduction of clinically unnecessary attendances may be supported by population education, although more substantial progress is likely to require investment in better social and welfare services and community development.

## Next steps to support research in this area

Even when one recognizes the conceptually distinct categories of avoidable attendances discussed above, it may not be easy to distinguish them in practice. For the full potential of these definitions to be realized, attention needs to be paid to how they can be operationalized using hospital records. Improvements in data collection are almost certainly required to facilitate more accurate identification of each distinct type of avoidable attendance. Furthermore, it is important to note that, just like ACSCs, the definitions we suggest will identify attendances which are *potentially* avoidable. There is a limit to what can be done with an algorithmic approach and we cannot know for certain whether an intervention would have avoided any particular attendance. Instead, once the drivers of demand that are common across all attendances have been accounted for, these measures will give important information on potentially avoidable demand for health care,^21^ signalling potential issues with the provision of other services within the system.

Most of the research on avoidable attendances in England focuses on what we describe as clinically divertible attendances. There are currently many approaches used to quantify these types of attendances using administrative data, and this is problematic because different definitions will yield different estimates of prevalence. A standardized method is therefore needed. Furthermore, the methods of identification previously used in the literature to identify clinically divertible attendances will also be capturing clinically unnecessary attendances. It is important that these two groups can be distinguished from one another because the type of care required (no clinical care vs. non-specialized clinical care) and associated potential policy solutions will be different. Making this distinction may not be easy and may require additional data collection in routine ED practice. Definitions of ACSCs currently used to study emergency admissions are based on International Classification of Diseases-10 (ICD-10) diagnosis codes. However, these are not routinely collected in EDs in England, making it difficult to straightforwardly apply this concept in the ED context. Again, further research is required in order to develop and test methods of measurement and data capture which are reproducible and acceptable to clinicians and patients.

## Discussion

Rising demand for emergency care is an issue facing healthcare systems around the world, with many implementing policies to target reductions in ED attendances. There is currently an implicit assumption that avoidable attendances are an issue to be tackled, but full understanding of the magnitude of the problem is lacking. Moreover, many policies fail to fully articulate the types of attendances that are targets for reductions or the underlying causal mechanisms by which these impacts are hypothesized to occur.

We have questioned the usefulness of the current broad concept of avoidable ED attendances and identified three key areas of inconsistency surrounding the concept in its current form. The current disparities in the terminology, definitions and methods of operationalizing the concept of avoidable emergency ED attendances are limiting progress in this area. We have proposed a refined classification based on the care that was required.

Despite more than a decade of policy interventions seeking to reduce demand for emergency care, reviews of the literature on such interventions find insufficient evidence to support effectiveness.^22^ Demand for care is based on the complex interaction between multiple factors, such as population characteristics, patient personal and social characteristics and health service delivery. Distinguishing between the different types of avoidable attendances will facilitate research into the factors driving variation of each and allow a more nuanced understanding of the mechanisms underlying each type of potentially avoidable attendance. High-quality qualitative work alongside quantitative exploration of causal associations based around these definitions will support the development of a more sophisticated understanding of the factors driving ED attendance, and the development of policy solutions which take account of the social and cultural factors affecting population behaviours. Whilst we accept that operationalizing our refined definitions of avoidable attendances may pose some practical challenges, we believe that they will provide a platform upon which such research can be built.

Whilst heterogeneity still exists within the three attendance categories we have proposed, classifying attendances according to the care that was required frames the issue in terms of potential solutions. Defining attendances in this way allows health system planners to more clearly identify what the underlying problems affecting the system are, and to subsequently design appropriate interventions to address these problems. For example, if most avoidable attendances are *clinically unnecessary,* the causes and therefore solutions mostly lie outside of the health care system and there is a limit to what could be achieved by changes to the organization or provision of healthcare services. If this were the case it would call into question the appropriateness of initiatives such as improving access to routine primary care appointments. However, if most are *clinically divertible* then health system planners need to ensure the availability of easily accessible, less specialized alternatives. Defining and measuring *clinically preventable* attendances will, as has been achieved with ACSCs for admissions, provide an indicator of primary/community care quality, as well as supporting the development of interventions to reduce overall system costs by improving prevention and management in the community.

The magnitudes of the different types of avoidable ED attendances will differ across countries, as the nature and availability of urgent care services varies considerably between health systems. The scope of each service, the availability of out-of-hours services and alternative emergency urgent care services will all alter the types of patients defined as divertible or preventable. The payment arrangements will also impact where patients choose to seek care. Furthermore, the policy solutions to deal with the different types of avoidable attendances may also differ between health systems. For example, in systems with no universal health coverage, reducing divertible attendances will require some attention to the insurance and other systems in place. Systems with universal coverage may wish to focus upon increased availability and signposting of services.

We have approached these concepts from the perspective of the health system. Research on the patient’s decision to attend the ED with minor problems suggests patient anxiety is a key driver of these types of attendances.^23^ A recent survey of the British public’s views about emergency care found that 36% of respondents prefer NHS services where no appointment was needed and 51% find it hard to get a GP appointment.^24^ Consideration of the patient’s perspective is therefore required when developing interventions to reduce demand, since this may be a barrier to the effectiveness of policies if alternatives are not acceptable to patients.

Whilst we did not follow systematic review methods, studies included in the literature review were identified from three systematic reviews on avoidable attendances, as well as searching forward citations of these reviews and the papers they included, to identify more recent studies. The aim of the search, and subsequent review, was to highlight the complexity of the issues involved and the lack of uniformity in the terminology, definitions and methods. It is unlikely that a more systematic search would change our conclusion that there is currently a lack of clarity and consistency.

Recognizing distinct categories of avoidable attendance and developing methods to operationalize these definitions will allow quantification of the magnitude of each type of attendance. This will provide a better understanding of whether avoidable attendances do pose as large a problem as is currently suggested and identify if it is worth implementing policies to try to reduce them. Furthermore, this will facilitate research into the drivers of each category of avoidable attendance and potential policy solutions and provide a refined metric for use in the subsequent evaluation. Future research should focus on the operationalization of these refined concepts in the first instance.
